# Supplementary material for: Identification and functional analysis of non-coding regulatory small RNA FenSr3 in Bacillus amyloliquefaciens LPB-18
Source: PeerJ. 2023 May 15;11:e15236. doi: 10.7717/peerj.15236 (PMC10194069; doi:10.7717/peerj.15236)
Supplement: Supplemental Information 4 [file peerj-11-15236-s004.zip › KO/CK-vs-T1_map/map04913.html]

KEGG PATHWAY: Ovarian steroidogenesis - Reference pathway


|  |  |
| --- | --- |
| **Ovarian steroidogenesis - Reference pathway** |  |

[
Pathway menu
| Organism menu
| Pathway entry
| Show description
| User data mapping
]

The ovarian steroids, 17-beta estradiol (E2) and progesterone (P4), are critical for normal uterine function, establishment and maintenance of pregnancy, and mammary gland development. Furthermore, the local effects that are essential for normal ovarian physiology are dependent on the endocrine, paracrine, and autocrine actions of E2, P4, and androgens. In most mammals (including humans and mice), ovarian steroidogenesis occurs according to the two-cell/two-gonadotropin theory. This theory describes how granulosa and theca cells work together to make the ovarian steroids. Theca cells respond to LH signaling by increasing the expression of enzymes necessary for the conversion of cholesterol to androgens, such as androstenedione (A) and testosterone (T). Granulosa cells respond to FSH signaling by increasing the expression of enzymes necessary for the conversion of theca-derived androgens into estrogens (E2 and estrone).

|  |  |  |
| --- | --- | --- |
| Reference pathway | 184% 150% 122% 100% 82% 67% 55% | 图片下载 |
